# The socioeconomic gradient and chronic illness and associated risk factors in Australia

**DOI:** 10.1186/1743-8462-1-8

**Published:** 2004-12-12

**Authors:** John D Glover, Diana MS Hetzel, Sarah K Tennant

**Affiliations:** 1 Public Health Information Development Unit (PHIDU), The University of Adelaide, South Australia, Australia 5005

## Abstract

**Objective:**

To examine the prevalence of major chronic diseases and their risk factors in different socioeconomic groups in the Australian population, in order to highlight the need for public policy initiatives to reduce socioeconomic inequalities in health.

**Methods:**

Data were provided by the Australian Bureau of Statistics (ABS) from the 2001 National Health Survey (NHS) for selected chronic diseases and associated risk factors. Conditions selected were those, which form the National Health Priority Area (NHPA) conditions (other than injury, which has not been included in this paper, with its focus on chronic disease); plus other 'serious' chronic conditions, in line with the classification developed by Mathers; and for which sufficient cases were available for analysis by socioeconomic status. Indirectly age-standardised prevalence rates were calculated by broad age group for Australia and for five groups of socioeconomic status; rate ratios were calculated to show variations in prevalence between these groups.

**Results:**

Significant socioeconomic inequalities were evident for many of the major chronic diseases; the largest was for diabetes mellitus (at ages 25 to 64 years); and for many diseases, there was also a strong, continuous socioeconomic gradient in the rates.

Circulatory system diseases (in particular, hypertensive disease) and digestive system diseases also exhibited a strong differential in the 25 to 64 year age group.

In the 65 years and over age group, the strongest inequalities were evident for mental and behavioural problems, diabetes (with a continuous socioeconomic gradient in rates) and respiratory system diseases.

A number of risk factors for chronic diseases, namely self-reported smoking, alcohol misuse, physical inactivity and excess weight showed a striking association with socioeconomic status, in particular for people who were smokers and those who did not exercise.

**Conclusion:**

This analysis shows that the prevalence of chronic disease varies across the socioeconomic gradient for a number of specific diseases, as well as for important disease risk factors. Therefore, any policy interventions to address the impact of chronic disease, at a population level, need to take into account these socioeconomic inequalities.

## Background

As in other developed countries, chronic diseases in Australia are major contributors to the extent of illness, disability and premature mortality in the population. They are estimated to make up the greatest proportion of the burden of disease, mental problems and injury for the population as a whole (about 80%), and for particular sub-population groups [1].

Chronic diseases are exemplified by having multifactorial aetiologies, including common disease risk factors and determinants; significant latency periods and protracted clinical courses; and are seldom cured completely [2,3]. Causal factors interact together at an individual and at a population level to determine the degree of disease burden and illness, and unhealthy risks can be passed on through families, communities, and populations following demographic gradients [4]. At different life stages, common risk factors and determinants include poor intra-uterine conditions; stress, violence and traumatic experiences; educational disadvantage; inadequate living environments that fail to promote healthy lifestyles; poor diet and lack of exercise; alcohol misuse and tobacco smoking [5,30]. Risk factors are also increasingly more prevalent in areas of low socioeconomic status and in communities characterised by low levels of educational attainment; high levels of unemployment; substantial levels of discrimination, interpersonal violence and exclusion; and poverty. There is a higher prevalence of such factors among Indigenous communities, and other socioeconomically disadvantaged Australians [5,6].

The inequalities in health observed across populations are many – some of them are inevitable and others, unnecessary and unfair. Those inequalities that are potentially avoidable are deemed 'inequitable' [7]. Despite significant medical advances and improved public health in recent decades, socioeconomically disadvantaged communities continue to suffer an unequal burden of illness, premature death and disability. Therefore, the study of socioeconomic inequalities in chronic diseases and conditions and in risk factors is important and necessary. This is particularly so, if we wish to develop more effective policy mechanisms for preventing and intervening earlier in the progression of chronic diseases and their associated risk factors across the diverse Australian population, and to reduce some of the existing health inequities.

### Our approach

There have been a number of studies published in Australia on socioeconomic inequalities in mortality from various chronic diseases and conditions. The earlier ones were analysed using information on occupation recorded on the death certificate [8-10]. An alternative approach has been to examine variations in mortality rates by grouping residential locations according to socioeconomic criteria. A number of such studies have documented substantial variations in mortality for different age groups [11-17].

However, to date, there have been fewer studies that have examined socioeconomic inequalities in chronic disease prevalence in those still living (analyses of hospital admissions for chronic conditions have been published, but not of prevalence) [15]. One of the earliest was undertaken by Broadhead, who analysed data on morbidity and social status from the 1977–78 Australian Health Survey (ABS), and found that men in lower status occupations tended to suffer a higher age-standardised rate of self-reported chronic conditions and days of reduced activity; the picture for women appeared less clear [18]. Lee et al found that low income males were more likely to report mental health problems, chronic symptoms and acute symptoms than their high income counterparts [19]. Similar findings were reported in other studies, and risk factors associated with chronic diseases also were also associated with low income [15,19-21].

The work of Mathers is significant for its systematic documentation of health inequalities among working aged Australians (25 to 64 years) in the late 1980s. He examined mortality, disability, disease groups, specific diseases, self-perceived health, risk factors, health service use and use of preventive screening services, using data from the 1989–90 National Health Survey (NHS) [15]. Mathers found that there were no clear gradients of chronic, recent or minor illness with level of socioeconomic disadvantage of area, although there were some specific health status indicators (self-reported health, reduced activity, unhappiness) and certain risk factors (inactivity, smoking and alcohol use) that were reported more frequently by those in the more disadvantaged quintiles [15].

Current information on inequalities in health other than mortality is limited in Australia because of a dearth of suitable data collections. However, the release of data from the 2001 NHS provides an opportunity to examine the prevalence of self-reported chronic disease in Australia and the way in which this impacts on different socioeconomic groupings within the population.

## Results

Information for a selection of chronic diseases is shown in Table 1. Diseases were included on the basis of either high prevalence or their contribution to the burden of disease.

**Table 1:** Inequality in prevalence of selected chronic diseases^1^, 2001

Age group (years) and chronic disease	Rate^2^	Rate ratio by quintile of socioeconomic disadvantage of area^3^				
		
		First	Second	Third	Fourth	Fifth
**0–14**^4^						
Mental and behavioural problems^5^	6 596	1.00	1.04	1.10	1.12	1.52**
Respiratory system	21 807	1.00	1.07	1.05	1.11	0.99
Asthma	13 363	1.00	1.10	1.12	1.25*	1.12
**15–24**						
Mental and behavioural problems^5^	10 284	1.00	1.02	0.97	1.08	1.28
Respiratory system	33 373	1.00	1.04	1.12	1.09	1.00
Asthma	16 263	1.00	0.82	1.14	1.02	1.00
Bronchitis/emphysema	1 701	1.00^6^	1.32^6^	1.66^6^	1.94^6^	1.97^6^
Musculoskeletal system^7^	19 088	1.00	1.11	1.00	1.08	0.94
**25–64**						
Diabetes mellitus	2 234	1.00	1.37	1.67*	1.72*	2.28***
Mental and behavioural problems^5^	11 093	1.00	1.05	1.20*	1.36***	1.67***
Circulatory system	17 491	1.00	1.04	0.97	1.15*	1.28***
Hypertensive disease	9 751	1.00	1.12	1.01	1.24*	1.54***
Respiratory system	32 964	1.00	1.00	0.99	0.99	1.01
Asthma	10 393	1.00	1.10	0.99	1.19*	1.14
Bronchitis/emphysema	3 429	1.00	0.97	1.14	1.55**	1.70***
Digestive system	8 074	1.00	1.03	1.07	1.12	1.37***
Musculoskeletal system^7^	39 840	1.00	1.10*	1.16***	1.16***	1.22***
**65 & over**						
Diabetes mellitus	8 981	1.00	1.13	1.14	1.52*	1.56*
Mental and behavioural problems^5^	7 222	1.00	1.21	1.62*	1.67*	1.56*
Circulatory system	56 592	1.00	1.09	1.06	1.10	1.19*
Respiratory system	31 442	1.00	1.03	0.87	0.95	1.22*
Musculoskeletal system^7^	63 669	1.00	1.02	1.06	1.03	1.08

^1^Survey respondents can report more than one disease.

^2^Rate is the number of persons per 100,000 population reporting the disease.

^3^The extent of any inequality is shown by the rate ratio, which expresses the ratio of the rate in each quintile to the rate in Quintile 1 (the most advantaged areas, with a rate ratio of 1.00); rate ratios differing significantly from 1.0 are shown with * p < 0.05; ** p < 0.01; *** p < 0.001.

^4^Information about these age groups were collected by proxy, using parental report.

^5^Information may be based on self-diagnosis, rather than diagnosis by a health practitioner.

^6^Indicates rate ratio based on estimates with a Relative Standard Error of between 25% and 50% and should be used with caution.

^7^Includes diseases of the connective tissue.

Source: National Health Survey, ABS 2002

The main findings are:

The largest differential between those in the most well off and those in the most disadvantaged areas was for diabetes mellitus at ages 25 to 64 years, with the prevalence in the most disadvantaged areas being just over two and a quarter times (a rate ratio of 2.28) the prevalence for the least disadvantaged; there is also a strong, continuous gradient in the rates, with the rate ratios in each of the third to fifth quintiles statistically significant.

There was a statistically significant differential of 67% at ages 25 to 64 years, with a strong, continuous gradient, in the prevalence of self-reported mental and behavioural problems across the socioeconomic gradient; differentials (also statistically significant) in the 0 to 14 year and 65 years and over age groups were 52% and 56%, respectively.

Circulatory system diseases (in particular, hypertensive disease) and digestive system diseases also exhibit a strong differential in the 25 to 64 year age group (statistically significant differentials of 28% and 54%, respectively).

In the 65 years and over age group, the strongest differentials were evident for mental and behavioural problems (a statistically significant 56%), diabetes (with a continuous gradient in rates, statistically significant in quintile3 four and five) and respiratory system disease (a statistically significant 22%).

Asthma accounted for almost two thirds of the rate of reporting of respiratory system disease in the 0 to 14 year age group, almost half in the 15 to 24 year age group, and for about a third of the rate in the 25 to 64 year age group.

The NHS also included data on a number of risk factors for chronic diseases, namely self-reported smoking, alcohol misuse, physical inactivity and excess weight. A number of these risk factors show a striking association with socioeconomic status, in particular for people who are smokers and those who did not exercise, with continuous gradients and highly elevated rates of statistical significance (Table 2). The differences in male and female rates are also of interest. It was only for underweight females, and for the risk factor of high-risk alcohol consumption by females, that the socioeconomic gradient was reversed.

**Table 2:** Inequality in prevalence of selected health risk factors, 18–64 years, 2001^1^

Health risk factors	Rate^2^	Rate ratio by quintile of socioeconomic disadvantage of area^3^				
		
		First	Second	Third	Fourth	Fifth
Current smokers - Male	30 582	1.00	1.40***	1.55***	1.71***	1.95***
- Female	24 009	1.00	1.29***	1.34***	1.48***	2.00***
- Persons	27 275	1.00	1.35***	1.45***	1.61***	1.96***
Alcohol – High risk - Males	6 976	1.00	1.09	1.26*	1.26*	1.45***
- Females	2 127	1.00	0.59*	0.94	0.76	0.87
- Persons	4 537	1.00	0.93	1.16	1.12	1.22*
Did not exercise - Males	28 772	1.00	1.20**	1.36***	1.52***	1.68***
- Females	28 220	1.00	1.19**	1.29***	1.35***	1.65***
- Persons	28 494	1.00	1.20***	1.32***	1.43***	1.66***
Underweight females	12 675	1.00	0.89	0.83*	0.72***	0.91
Overweight/obese - Males	54 701	1.00	1.09*	1.11*	1.04***	1.00
- Females	37 004	1.00	1.09	1.21***	1.16**	1.17**
- Persons	45 798	1.00	1.09**	1.15***	1.09**	1.06

^1^Survey respondents can be shown under more than one type of risk factor.

^2^Rate is the number of persons per 100,000 population estimated to be at risk from the health risk factor.

^3^The extent of any inequality is shown by the rate ratio, which expresses the ratio of the rate in each quintile to the rate in Quintile 1 (the most advantaged areas, with a rate ratio of 1.00); rate ratios differing significantly from 1.0 are shown with * p < 0.05; ** p < 0.01; *** p < 0.001.

Source: National Health Survey, ABS 2002

It is important to note that the inequalities reported above relate to the health of those people living in a geographic area and to the overall level of socioeconomic disadvantage of that area. Most areas will contain varying levels of individual socioeconomic disadvantage and, to the extent that the poorer health is associated with individual economic circumstances and living conditions rather than communal environment, the inequalities will understate the true differences in health status according to socioeconomic disadvantage [15].

Furthermore, there are limitations to the use of area-based measures of SES. Due to misclassification error (i.e. ascribing area-SES to individuals), estimates of difference across the quintiles will be smaller than if data on individual-level measures of SES were used [28]. Thus, chronic disease inequalities in the wider population by SES are likely to be larger than those reported in this study. In addition, the exclusion of the 'sparsely settled' areas of Australia in NHS data collection results in the omission of data from a high percentage of Indigenous people, who are the population group with the poorest health.

## Discussion

Our analysis indicates that socioeconomic inequalities in the prevalence of chronic diseases and their concomitant risk factors are evident across the Australian population. However, the diseases with substantial disparities across the socioeconomic quintiles are different, for different stages in the life course. Although these results cannot be directly compared with those of previous studies, because of definitional and methodological differences, the recurring finding of inequalities for chronic disease morbidity and risk factor prevalence across the socioeconomic gradient remains a significant concern.

The burden in the Australian population attributable to socioeconomic inequality is large, and has far-reaching implications in terms of unnecessary disability and suffering, the loss of potentially economically productive members of society, and increased costs for the health and social care systems [35]. Despite the expenditure of millions of dollars to prevent and reduce the prevalence of chronic diseases and their risk factors, these inequities have persisted. However, the situation in Australia is by no means unique, for inequalities in these diseases and their risk factors have been observed for most of the developed countries in which they have been studied [26].

What should we be doing differently? There is a growing body of knowledge that will help to provide direction for developing policies to reduce inequities across the population. The socioeconomic environment is a powerful and potentially modifiable factor, and public policy is a key instrument to improve this environment, particularly in areas such as housing, taxation and social security, work environments, urban design, pollution control, educational achievement, and early childhood development [34].

However, attention must be paid to the nature of any action that is taken, to ensure that social and economic inequalities are not increased. Some programs, by their very success, can increase inequality by widening the gap between groups in the population; for example, such programs may be more attractive to those who are already healthier, or not as effective for certain groups with poorer health, less education or more stressful lives. In one smoking cessation initiative, it was found that the prevalence of smoking decreased predominately in those adults with higher education, thus increasing the existing difference with those who were more disadvantaged [37]. While smoking prevalence in Australia has reduced considerably over the last 20 years, attributes such as lower education and occupational status, unemployment, rented housing, and living in disadvantaged areas reflect a higher probability of reporting tobacco expenditure [32]. As a result, the tax revenue from the sale of tobacco products is being disproportionately drawn from the poorest households and represents a greater proportion of their household budget [32].

It is also evident that the ways in which systems such as education and health are delivered and structured can increase existing inequality. For example, schooling can be a way of addressing inequality and also a way of reproducing it. It has been suggested that there are two goals for a social justice program in education: to work to eliminate the contribution that the education system makes to the production over time of social inequality in general; and to maximise the positive contributions that the education system makes to reducing social inequality [33]. Therefore, different approaches and mixes of policies and programs must be mounted to address inequalities. These approaches may include more precise targeting, but also greater attention to community-based dimensions of 'interdependence' between individual behaviours, key determinants, and community and institutional resources.

Policy-makers who wish to address socioeconomic inequalities in health may favour one of the following approaches. Some view the impact of socioeconomic disadvantage on those groups with the poorest health in the population, such as Aboriginal people and Torres Strait Islanders, as the priority policy goal. Others identify the gap between the health of those groups at the outer ends of the socioeconomic hierarchy (those with the poorest health and those with best health), and see the narrowing of the gap as the goal. Others prefer to focus on the socioeconomic gradient in health that runs across the whole population [31].

Graham has identified that the last approach widens the policy debate in three ways [31]. Firstly, it looks for the causes of health inequality in the systematic differences in life chances and opportunities, living standards and lifestyles that are associated with people's unequal positions across the socioeconomic hierarchy, and for the pathways through which they influence health [31,36]. Secondly, as a result, addressing health inequalities becomes a population-wide goal that includes every citizen [31]. Thirdly, 'reducing health gradients' provides a more comprehensive policy approach: one that encompasses 'remedying disadvantages' and 'narrowing health gaps' within the broader goal of 'equalising health chances across all the socioeconomic groups' [31].

She also observes that, "improving the health of poor groups and improving their position relative to other groups are necessary elements in a strategy to reduce the socioeconomic gradient. However, neither is sufficient on its own. To reduce the socioeconomic gradient, health in other socioeconomic groups also needs to improve at a faster rate than in the highest socioeconomic group. Thus, policies to ameliorate health disadvantage, to close health gaps and to reduce health gradients need to be pursued together, and not at the expense of each other" [31].

There is also an urgent need to make health inequalities a research priority for each stage of the life course – not just to monitor the size and extent of the disparities but also to undertake research that will find preventive approaches and further policy interventions that will be effective in reducing them, and that are likely to be implemented by governments and communities.

## Conclusions

Clearly, any moves to address the impact of chronic disease at a population level must take into account socioeconomic inequalities in prevalence. More research is needed to determine which approaches are effective and why others have failed to have the desired impact, particularly for those who are from socioeconomically disadvantaged areas. Finally, although rates are generally highest at the oldest ages, the development of risk factors for many chronic diseases occurs early in life, and thus, it is essential those health inequities are addressed right across the life course.

## Methods

### Data sources

The ABS conducts the National Health Survey (NHS) on a regular basis, most recently in 2001 [22]. The NHS collects information from approximately 26,900 people from all States and Territories living in private dwellings, selected at random using a multi-stage area sample of private dwellings. The survey is undertaken across much of Australia, but excludes the 'sparsely settled' areas, which comprised less than 1% of the non-Indigenous population and 25% of the Indigenous population at the 2001 Census: a separate Australia-wide survey of the health of Indigenous people, also conducted in 2001, surveyed these sparsely settled areas.

The survey includes self-reported details of health conditions (both acute and long term) and major risk factors, as well as demographic and socioeconomic information about the survey respondent. Respondents were asked if they had been told by a doctor or nurse that they had asthma, cancer, heart and circulatory conditions, and/or diabetes. These conditions, together with injuries and mental health, form the NHPAs [27]. However, for long term mental health problems, respondents were not asked whether they had been told by a doctor or nurse that they had any mental health problems; thus, the responses may be based on self-diagnosis, rather than diagnosis by a health practitioner [22]. Respondents were also asked a series of questions about other specific, non-NHPA, conditions, covering eye and sight problems, ear and hearing problems, and arthritis, rheumatism and gout. They were then shown a series of three prompt cards (two with conditions listed, while the third contained more general descriptions of condition types) and asked whether they had any of the conditions shown or conditions similar to those shown or described. In each of these cases, details were recorded for conditions reported as current at the time of the survey; respondents were also asked whether the condition had lasted, or was expected to last, for six months or more. Information was gathered directly from individuals aged 15 years and older. For children up to the age of 15 years, information was provided by proxy, from a parent or guardian.

The particular conditions for which data were requested from the ABS for this analysis were:

the NHPA conditions (other than injury, which has not been included in this paper, with its focus on chronic disease); plus other 'serious' chronic conditions, in line with the classification developed by Mathers [15]; and for which sufficient cases were available for analysis by five groups of socioeconomic disadvantage of area (see below for details of the way these groups were constructed).

The risk factors used by the ABS were those identified for the NHPA conditions [27].

The ABS has coded conditions reported by respondents to output disease categories based on ICD-10. Conditions described as 'chronic' in this article include those long-term conditions reported in the NHS, which are commonly recognised by health practitioners as chronic diseases [23]. The risk factor for 'high risk due to alcohol' reflects the National Health and Medical Research Council's risk levels for harm in the long term from alcohol consumption [24]. The risk factors for overweight and underweight were calculated from self-reported height and weight information and grouped to reflect World Health Organization (WHO) guidelines [25].

Given the policy importance of the NHPAs, the 2001 NHS questionnaire underwent significant revisions to more precisely capture information on several of the NHPAs. Consequently, while the quality of the information on NHPAs has been improved from the 1995 NHS, the degree of comparability with previous surveys has been somewhat compromised for many of the major health conditions. Some specific conditions (e.g., diabetes) appear to be comparable between the 1989–90 and 2001 surveys, however, for most groups of conditions based on ICD chapter headings (e.g., all circulatory) the ABS advise that the combined effect of major conceptual changes as well as major classification changes between the 1989–90 and 2001 surveys would make direct comparisons very difficult. This analysis is therefore restricted to the 2001 data.

### Measurement of socioeconomic status

The socioeconomic status (SES) of the address of residence of each survey respondent is available at the Census Collection District (CD) level and was added to the NHS file, as was the quintile of socioeconomic disadvantage of area into which that CD fell at the Census. The measure used to allocate CDs to quintiles was the 1996 Census Index of Relative Socio-Economic Disadvantage (IRSD). The IRSD is one of five Socio-Economic Indexes for Areas produced by the ABS using Principal Components Analysis. It summarises information available from variables collected in the 1996 Population Census including those related to education, occupation, and income. The variables are expressed as percentages of the relevant population.

The NHS records were aggregated to the quintiles derived from the Census data, where Quintile 1 comprises the CDs with the highest IRSD scores (highest socioeconomic status, or most advantaged, areas) and Quintile 5 comprises the CDs with the lowest IRSD scores (lowest socioeconomic status, or most disadvantaged areas). Each quintile comprises approximately 20% of CDs.

The ABS produced the estimates of the number of people with chronic diseases and risk factors by quintile. The method used resulted in the production of quintiles of varying sizes, ranging from 17.4% of the population in Quintile 5 (most disadvantaged areas) to 22.8% in Quintile 2 and 21.1% in Quintile 1 (most advantaged areas). This is a differential of over five percentage points between Quintile 5 and Quintile 2, or 1,027,030 fewer people in the most disadvantaged areas when compared with Quintile 2 (and 708,980 fewer in Quintile 5 than in Quintile 1). The effect of this lack of precision on the results by quintile is not known. Although, in part, the difference arises as a result of the method used (that is, that the quintiles are based on the Census population, and applied to the NHS population which has a different profile), it may also reflect different response rates to the survey from different socioeconomic groups.

The NHS includes other measures that could potentially be used to measure socioeconomic status. For example, income reporting in ABS surveys is known to be incomplete, in particular for low income groups and, in the 2001 NHS, income was only collected for the respondent and their spouse. Information on education is also available, but a single measure has yet to be agreed. Problems in using education include that it is typically completed early in adulthood; it captures neither differential on-the-job training and other career investments made by individuals with similar levels of formal schooling, nor the volatility in economic status during adulthood that has been shown to have adverse implications for health [29]. The age structure of the population may also influence the indicator: an older population generally has lower education levels than a younger population due to improved access to education over time.

### Analysis

Chronic disease and risk factor rates are expressed as rates per 100,000 population, indirectly age-standardised. The rates were also calculated by age although, given the small sample size of the NHS, only broad age groups (0–14 years, 15–24 years, 25–64 years and 65 years and over) were available. The standard population and quintile populations are the weighted 2001 survey populations from the NHS. The extent of any differential between the quintiles is shown by the rate ratio, which expresses the ratio of the rate in each quintile to the rate in Quintile 1 (the most advantaged areas, with a rate ratio of 1.00).

## Competing interests

The author(s) declare that they have no competing interests.

## Authors' contributions

JG conceived the study, and was responsible for its design and coordination. ST participated in the design of the study and performed the statistical analysis. DH participated in the drafting of the manuscript. All authors read and approved the final manuscript.
